# Development of an exercise intervention for the prevention of musculoskeletal shoulder problems after breast cancer treatment: the prevention of shoulder problems trial (UK PROSPER)

**DOI:** 10.1186/s12913-018-3280-x

**Published:** 2018-06-18

**Authors:** Helen Richmond, Clare Lait, Cynthia Srikesavan, Esther Williamson, Jane Moser, Meredith Newman, Lauren Betteley, Beth Fordham, Sophie Rees, Sarah E. Lamb, Julie Bruce, on behalf of the PROSPER Study Group, Julie Bruce, Sarah E. Lamb, Esther Williamson, Ranjit Lall, Stavros Petrou, Alastair Thompson, John Williams, Helen Richmond, Clare Lait, Sophie Rees, Pankaj Mistry, Alastair Canaway, Bruno Mazuquin

**Affiliations:** 1 Warwick Clinical Trials Unit, Division of Health Sciences, University of Warwick, Coventry, CV4 7AL UK; 2 Gloucestershire Care Services NHS Trust, 1010 Gloucester Business Park, Pioneer Avenue, Brockworth, Gloucester, GL 3 4AW UK; 3 Botnar Research Centre, Nuffield Department of Orthopaedics Rheumatology & Musculoskeletal Sciences, University of Oxford, Windmill Road, Oxford, OX3 7LD UK; 4 Physiotherapy Research Unit, Oxford University Hospitals NHS Foundation Trust, Nuffield Orthopaedic Centre, Windmill Road, Oxford, OX3 7HE UK

**Keywords:** Breast cancer, Shoulder dysfunction, Shoulder exercises, Physical activity, Behavioural support strategies, Upper limb, Postoperative complications, Physical therapy, Physiotherapy, Exercise therapy, Supported care

## Abstract

**Background:**

Musculoskeletal shoulder problems are common after breast cancer treatment. There is some evidence to suggest that early postoperative exercise is safe and may improve shoulder function. We describe the development and delivery of a complex intervention for evaluation within a randomised controlled trial (RCT), designed to target prevention of musculoskeletal shoulder problems after breast cancer surgery (The Prevention of Shoulder Problems Trial; PROSPER).

**Methods:**

A pragmatic, multicentre RCT to compare the clinical and cost-effectiveness of best practice usual care versus a physiotherapy-led exercise and behavioural support intervention in women at high risk of shoulder problems after breast cancer treatment. PROSPER will recruit 350 women from approximately 15 UK centres, with follow-up at 6 and 12 months. The primary outcome is shoulder function at 12 months; secondary outcomes include postoperative pain, health related quality of life, adverse events and healthcare resource use. A multi-phased approach was used to develop the PROSPER intervention which was underpinned by existing evidence and modified for implementation after input from clinical experts and women with breast cancer. The intervention was tested and refined further after qualitative interviews with patients newly diagnosed with breast cancer; a pilot RCT was then conducted at three UK clinical centres.

**Discussion:**

The PROSPER intervention incorporates three main components: shoulder-specific exercises targeting range of movement and strength; general physical activity; and behavioural strategies to encourage adherence and support exercise behaviour. The final PROSPER intervention is fully manualised with clear, documented pathways for clinical assessment, exercise prescription, use of behavioural strategies, and with guidance for treatment of postoperative complications. This paper adheres to TIDieR and CERT recommendations for the transparent, comprehensive and explicit reporting of complex interventions.

**Trial registration:**

International Standard Randomised Controlled Trial Number: ISRCTN 35358984.

## Background

Advances in the early detection and improved treatment of breast cancer have resulted in increased survival after diagnosis, resulting in many more women living with the consequences of cancer treatment [1]. Invasive therapy to the chest and axilla can lead to shoulder and upper body problems, resulting in reduced range of shoulder movement, muscle weakness, pain and functional limitations [2, 3]. There is some evidence that postoperative exercise may improve shoulder function in women at higher risk of shoulder problems after breast cancer surgery; however, uncertainty remains over the optimal content, timing and cost-effectiveness of exercise interventions [2]. Additionally, previous trials investigating the effectiveness and safety of postoperative exercise have methodological weaknesses including small sample sizes, limited duration of participant follow-up, lack of inclusion of important functional outcomes and failure to describe trial interventions adequately [2]. This weak evidence base resulted in the National Institute for Health Research (NIHR) commissioning the UK PROSPER trial (PRevention Of Shoulder ProblEms tRial); a randomised controlled trial (RCT) evaluating the clinical and cost effectiveness of early exercise in women with newly diagnosed breast cancer.

The aim of this paper is to comprehensively describe the PROSPER exercise intervention to be tested in the PROSPER trial, and to detail the processes underpinning the intervention development. Insufficient description of trial interventions hampers replication in future studies and can delay implementation into routine clinical practice for interventions found to be effective. As per Medical Research Council (MRC) guidance for the development and evaluation of complex intervention trials and recent calls for improved reporting of trial interventions, we followed the Template for Intervention Development and Replication (TIDieR), and considered the recently published Consensus on Exercise Reporting Template (CERT) for comprehensive reporting of exercise interventions [4–6].

## Methods

### The PROSPER trial

The UK NIHR Health Technology Assessment (HTA) Programme commissioned a large-scale, multicentre, pragmatic randomised controlled trial to investigate the clinical and cost-effectiveness of early exercise to prevent musculoskeletal shoulder problems in women at high risk of developing shoulder problems after breast cancer surgery. The PROSPER trial is registered internationally (ISRCTN: 35358984), has ethical approval and the full protocol has been published [7]. In brief, the trial will recruit 350 women from approximately 15 National Health Service (NHS) breast cancer units across England. Justification for the sample size calculation is provided in the detailed protocol, however the trial is powered to detect a 7-point difference on the Disability of the Arm, Shoulder and Hand (DASH) questionnaire [7]. Women at high risk of shoulder problems are eligible. High-risk criteria include one or more of the following: axillary node clearance (ANC) regardless of type of breast surgery, radiotherapy to the axilla or supraclavicular area, pre-existing shoulder problems or a body mass index (BMI) of ≥30. The primary outcome is shoulder function, measured using the DASH. Trial participants are randomised to receive either best practice usual care or usual care plus the PROSPER intervention.

### Overview of intervention development process

**Fig. 1:**
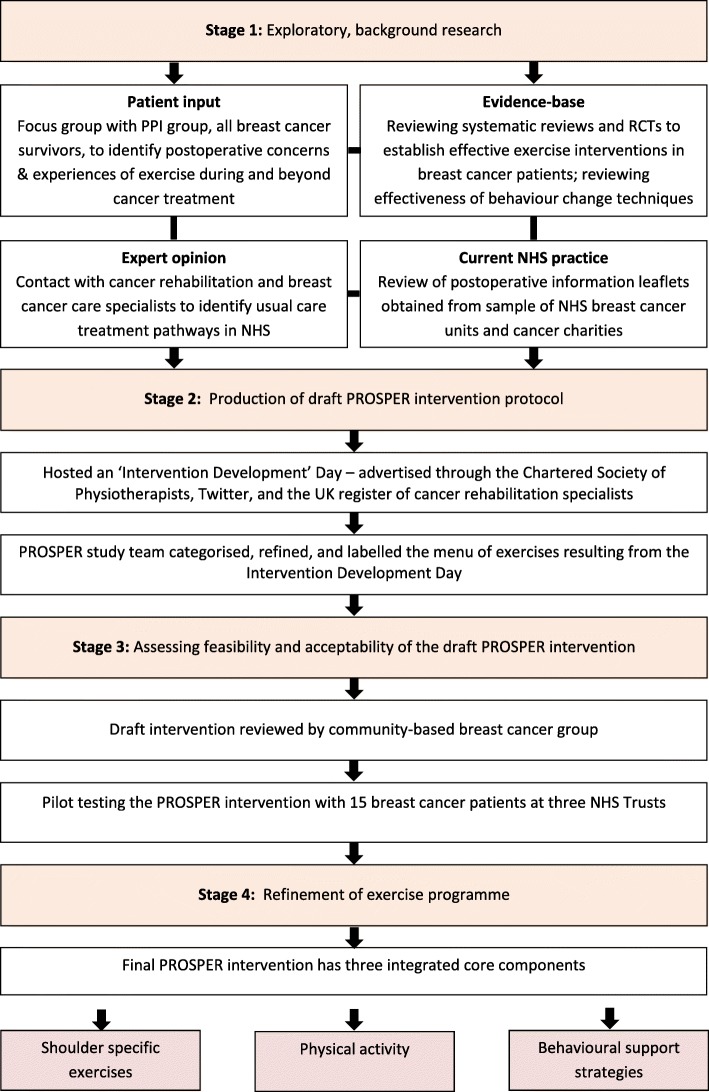
Overview of intervention development process

The PROSPER intervention and patient materials were developed and tested over a 12-month period. We selected intervention components based on recent systematic reviews and clinical guidelines. We augmented this by eliciting opinions from clinical experts within the field of cancer rehabilitation and health psychology. Figure 1 provides an overview of the multiple processes undertaken. We describe the key processes and findings from each phase that led to the final intervention.

#### Stage 1a: Exploratory work with women treated for breast cancer

Our four Patient and Public Involvement (PPI) representatives, all of whom had undergone breast cancer treatment, attended a formal 2-h meeting to explore views about the proposed trial and their experiences of postoperative exercise (facilitated by EW and JB). Our patient representatives were identified from a cancer advocate group (http://www.independentcancerpatientsvoice.org.uk/), a previous breast cancer study [8], and from a personal contact of the team. Women reported feeling generally unsupported after hospital discharge and described their experiences of persistent complications and self-management strategies. Two women suffered from ongoing lymphoedema, one had shoulder stiffness and pain. They advised that any programme should be adaptable and flexible to allow for prolonged treatment schedules and cancer-related fatigue.

#### Stage 1b: Establishing components for inclusion in the draft PROSPER intervention

We searched the literature for trials of exercise interventions following breast cancer treatment to aid selection of the exercises for the draft PROSPER intervention. We considered the content, timing, duration and setting of exercises tested within clinical trials. A systematic review published in 2010 investigated the effectiveness of exercise interventions in preventing, minimising or improving upper-limb dysfunction due to breast cancer treatment [2]. This review included 24 trials and classified exercise type as active, active-assisted, passive range of movement, manual stretching, active stretching, and resistance exercises. Only six of these 24 trials compared postoperative exercise to usual care and all had small sample sizes (from 27 to 115 participants). Since publication of this review, we identified an additional 8 trials of postoperative exercise which varied in terms of intervention duration (7 days to 6 months), type of exercise (from passive shoulder ROM to Yoga), dose (once/week to 3–4 times/day), and mode (group/individual; home based/in hospital setting) [9–16]. All trials had mixed findings in relation to the effectiveness of the exercise interventions on shoulder range of movement (ROM) and strength.

#### Active and active-assisted ROM exercises

Women are at risk of developing restricted shoulder ROM after surgery and radiotherapy; restrictions to shoulder flexion, abduction, and abduction with external rotation are common [11, 17]. Damage to the lymph transport system can occur after surgery or radiotherapy, placing women at increased risk of developing secondary lymphoedema. ROM exercises have important physiological benefits after treatment including: (i) improved synovial fluid drainage and lymphatic flow through activation of a physiological mechanism called the trans-synovial pump and (ii) maintenance of blood and lymphatic flow to the joints and soft tissues [18]. ROM exercises may prevent shortening and weakness of the surrounding muscles and connective tissues that can occur following immobilisation after surgery [16, 19, 20]. Given the importance of ROM for regaining shoulder function, encouraging lymphatic flow and preventing muscle shortening, active-assisted and active shoulder ROM exercises were considered essential for inclusion in the draft PROSPER intervention.

In terms of timing of exercise delivery, the McNeely [2] systematic review included 10 trials comparing early active and/or active assisted ROM exercises (started from 1 to 3 days after surgery) versus delayed ROM exercises (≥4 days after surgery) after breast cancer surgery . Early ROM had beneficial effects on shoulder flexion and abduction in the short and long term without increased risk of seroma, delayed wound healing, wound aspiration, postoperative pain, or lymphoedema [2]. However, participants randomised to early ROM exercise were significantly more likely to have increased wound drainage volume and wound drainage duration by 1 day, although the clinical significance of this is unclear [2]. A recent trial found that shoulder ROM above 90 degrees from the first postoperative day resulted in a significantly greater risk of lymphoedema compared to restricting shoulder ROM to below 90 degrees for the first week (risk ratio (RR) 2.7, 95% confidence interval (CI) 1.1, 6.3) [13]. Thus, we opted to restrict shoulder ROM to 90 degrees for the first 7 days after surgery.

#### Stretching exercises

Surgery and radiotherapy can result in scarring and soft tissue injury causing tightening and contracture of muscles and connective tissue across the shoulder and chest area [20]. Stretching plays a key role in connective tissue remodelling and the production of collagen in response to injury [21]. Stretching can prevent negative physiological adaptations to the muscle spindles, the stretch reflex and proprioceptors, and prevent shortening of muscle fibres [22]. Previous breast cancer trials have focused on stretching the pectoralis muscle group [2, 11, 19]. While these studies have had mixed results in terms of improving shoulder ROM, there is no evidence that stretching the pectoralis muscle increases the risk of lymphoedema. Pectoralis muscle flexibility is essential for performance of many upper limb functional activities as well as to maintain the extended arm position required for radiotherapy. Therefore, we included a daily ‘stretch and hold’ exercise for the pectoralis muscles in the draft PROSPER intervention.

#### Strengthening exercise

Loss of muscle strength negatively affects activities of daily living and quality of life, and can increase fatigue, risk of comorbidity, and mortality in any population group [23, 24]. Breast cancer is more common in older women and muscular strength declines with age due to a gradual loss of muscle fibre size and number [25]. Compounding this, cancer treatment can significantly reduce isometric/isokinetic strength capacity and muscular endurance compared to healthy controls [24, 26]. Muscle function decline in cancer populations is associated with higher all-cause mortality, poorer quality of life and increased fatigue and pain [24]. Targeted strength training can lead to significant improvements in both muscle mass and strength, can improve insulin action, bone density, and energy metabolism, all of which can be problematic in breast cancer patients [27, 28]. Previous studies have shown that individually tailored upper limb strength training can significantly improve shoulder function and strength without increased risk of lymphoedema in breast cancer patients [27]. We therefore included individually tailored and progressive strengthening exercises in the draft PROSPER intervention.

#### General physical activity

Systematic reviews provide strong evidence that physical activity (PA) during and after cancer treatment is safe, can improve survival, reduce recurrence, and improve cancer-related side effects such as fatigue, anxiety and depression [29–31]. Despite the known benefits, the majority of individuals do not meet national PA guidelines during or after cancer treatment [32]. Courneya [33] estimated that only 20% of individuals achieve pre-diagnosis PA levels after cancer treatment. Frequently cited barriers include lack of support and fears over safety [34]. We included PA as a core component of the draft PROSPER intervention, following the American Cancer Society guidelines which recommend that cancer patients should complete at least 150 min of moderate activity and at least two sessions of strength training per week [34, 35].

#### Behaviour change strategies

Our patient representatives emphasised the importance of a self-management approach to postoperative rehabilitation. Adherence to any self-management intervention is essential to achieve physiological benefits. However, there are numerous barriers to breast cancer patients engaging in exercise, particularly during active treatment [36]. Physical and emotional barriers include pain, fatigue, nausea, fear of recurrence, personal beliefs and attitudes such as self-efficacy and motivation, while external barriers include the availability of support, and practical challenges of finding time for PA [34, 36, 37]. The National Institute for Health and Care Excellence (NICE) guideline ‘Behaviour Change: individual approaches’ (2014) recommend that behavioural strategies should be incorporated into any self-management intervention aimed at changing behaviour [38].

The NHS Health Trainer Manual, developed by behaviour change experts, is a widely used evidence-based practical guide detailing strategies for the promotion of positive health behaviour change [38]. We selected behaviour change techniques to meet the needs of the eligible PROSPER population [39]. We also aimed to increase motivation to exercise and encourage adherence by promoting a Motivational Interviewing (MI) style of communication between physiotherapists and trial participants. MI is an effective technique for facilitating change in lifestyle behaviours, such as weight loss or physical activity, and for addressing the psychosocial needs of cancer survivors [40]. Key strategies for behaviour change and MI were included within in the draft PROSPER intervention.

### Stage 2: Production of draft PROSPER intervention protocol

#### Stage 2a: Consensus meeting - intervention development day

We hosted a consensus meeting at University of Warwick Clinical Trials Unit for cancer rehabilitation specialists, upper limb physiotherapists and patient representatives. The day comprised a series of presentations and workshops to review exercises reported in the literature, those used in clinical practice, and to review printed information leaflets from a sample of UK breast cancer units. Workgroups focused on discussions about the key exercises for inclusion within the PROSPER exercise programme. Exercise dose (Frequency, Intensity, Time and Type: FITT), and rationale for exercise progression and regression were discussed. We also considered practical issues of timing and feasibility of delivery within the busy NHS setting. At the closure of the meeting, we had a long menu of 44 different upper limb exercises for potential inclusion in the draft PROSPER intervention.

Rehabilitation experts participating in our workgroups highlighted the need to include manual therapy techniques for women presenting with scar tissue tightness and cording. Women with cording are often referred for treatment because they cannot achieve the correct upper limb position for radiotherapy, leading to treatment delays. Cording, or axillary web syndrome, is a painful complication of breast cancer treatment that can severely restrict shoulder ROM and function. Whilst manual therapy is anecdotally reported as an effective treatment for cording, the evidence-base is weak and inconclusive [12, 41]. Given the widespread use of these manual therapies in clinical practice, we opted to include two basic manual therapy techniques in the draft PROSPER intervention; these could be used if soft-tissue restriction was identified as a barrier to undertaking exercise.

After the consensus day, we refined the longer exercise menu by classifying each exercise according to movement direction (e.g. flexion, abduction, external rotation with abduction etc.) and removing exercises with similar or overlapping movements. We applied patient friendly terminology (such as ‘The Woodchopper’ which involved a combination of shoulder abduction and external rotation) and a simple colour-coded framework to the exercises targeting forward (flexion), sideways (abduction) and open chest (combined abduction and external rotation) shoulder ROM. This classification provided a simple structure for both physiotherapists and participants.

#### Stage 2b: Qualitative interviews

The next iteration of the draft intervention was assessed using qualitative semi-structured interviews with seven women treated for breast cancer. Further modifications included changes to the terminology of participant exercise folders from ‘Your Exercises’ to ‘Your Physiotherapy Folder’. Women felt it was more motivational to follow a programme underpinned and prescribed by trained physiotherapists; they also felt the term ‘exercise’ would be off-putting for some. Exercise frequency was reduced from three times to twice-daily to reduce burden and encourage adherence. Women preferred our photographs of ‘real’ women doing exercises rather than cartoon illustrations, as used in many NHS information leaflets and cancer charity materials.

### Stage 3. Assessing feasibility and acceptability of the draft PROSPER intervention

#### Community based breast cancer support group

The almost finalised version of intervention materials were reviewed by women attending a community based breast cancer support group, some of whom were recently diagnosed whereas others had completed treatment months or years ago. Overall, women were very positive and the only recommendation was that lymphoedema should be described in more detail.

#### Pilot study

Any healthcare intervention must be feasible for delivery within busy NHS clinical environments. We tested pragmatic implementation by piloting the draft PROSPER intervention with 15 women newly diagnosed with primary breast cancer from three hospital sites. This enabled further refinement of intervention content and paperwork. We revised treatment pathways and algorithms for the management of postoperative complications including pain, cording, wound infection, lymphoedema, and cancer-related fatigue.

### Stage 4. The final PROSPER intervention

The PROSPER intervention aims to improve shoulder function through an early, progressive, home-based exercise programme with integrated behavioural support strategies (Table 1). Trained NHS physiotherapists working in any hospital setting can deliver the intervention. To standardise prescription and delivery, two physiotherapists from each site will be invited to attend a PROSPER intervention training session covering intervention theory and practice (mean duration 4.5 h). Each physiotherapist will be provided with a comprehensive reference manual detailing the theoretical rationale for the intervention, as per MRC recommendations for complex interventions [4].

**Table 1:** Overview of PROSPER exercise intervention, as per TIDIER Criteria

TIDieR Items	Description
Brief Name	PROSPER (**PR**evention **O**f **S**houlder **P**robl**E**ms T**r**ial)
Why	Breast cancer treatments can affect the muscles, nerves, and lymphatic vessels in the shoulder and upper body, leading to reduced range of movement, muscle weakness, pain, and reduced upper limb function. Structured exercise programmes, started within days or weeks from surgery, may improve shoulder movement, strength, and function.
What	A physiotherapy-led 12-month exercise programme.
*Materials: Participants*	Every trial participant in the exercise arm is given “Your Physiotherapy Folder”, a small A5 folder containing a detailed description of all exercises, advice about surgical recovery, physical activity, postoperative complications and returning to daily activities. It also contains an exercise diary, a goal-setting sheet with a contract, and a ‘hurdles/facilitators’ brainstorming sheet. Participants are provided with resistance bands (Thera-band© tubing) for strengthening exercises and can be provided with protective goggles if this is a local NHS Trust policy.
*Materials: Physiotherapists*	Each PROSPER physiotherapist is provided with a comprehensive training manual (A4 ring binder and flexible bound copy), a participant folder and copy of training presentation slides. Box files are given containing a selection of resistance bands and supplies of paperwork such as treatment logs and instruction laminates.
*Procedures*	All participants follow usual care for the first 7 days after surgery (restricting shoulder movement to 90 degrees of flexion and abduction). For intervention participants, the first physiotherapy appointment is at **7–10 days** after surgery. The assessment includes previous medical history, shoulder range of movement, shoulder strength (from 4 weeks), posture check, observation of wound and screening questions for pain and lymphoedema. Participants are provided with a folder (‘Your Physiotherapy Folder’), from which the physiotherapist and the participant jointly select exercises based on the assessment and participant preference. Physiotherapists use **motivational interviewing** techniques to help each participant to set goals, explore confidence to exercise, problem-solve any hurdles and to facilitate ongoing motivation and exercise adherence at subsequent sessions. All exercises are detailed in Fig. 2. Any exercises prescribed to the patient should be performed at home.
Who provides	NHS physiotherapists from various backgrounds, including musculoskeletal rehabilitation, women’s health and surgical care. Physiotherapists have varying experience of oncology rehabilitation, ranging from limited to extensive clinical experience. All physiotherapists receive 4–5 h of intervention training.
How	Three individual face-to-face appointments and up to three individual flexible appointments delivered either face-to-face or by telephone.
Where	Clinic appointments are mostly located in physiotherapy outpatient clinics within secondary care UK NHS Trusts. The home exercise programme is conducted independently at the participant’s home.
When	Contacts: 3 face-to-face appointments at recommended time points: 7–10 days, 4–6 weeks, and 12 weeks after surgery. Participants can have an additional three flexible appointments at any time, either via face-to-face or by telephone. The first appointment is 60 min, with all subsequent appointments lasting 30 min.
How much	See Table 3.
Tailoring	The intervention can be individually tailored to each participant: • Selection of starting exercises is a joint-decision making process, based on the physiotherapist’s assessment and participant preference. • Exercise progression (frequency, level, resistance, repetitions, and sets) is a joint-decision making process, depending on current progress, level of pain and ability. • Type and level of physical activity will vary by participant. • Number, timing and mode of the three additional appointments is flexible. • The integrated behavioural strategies may feature more prominently for patients with low confidence and motivation to exercise. Identifying and problem solving barriers to exercise will be highly specific and individualised. • Optional use of manual therapy for cording and additional exercises for specific issues such as fist pumps for lymphoedema.
Modifications	The intervention was modified after qualitative interviews and piloting. Key changes included: (1) change of name from “Your Exercise Folder” to “Your Physiotherapy Folder”; (2) reduction in number of exercises within longer menu; (3) change in terminology of “barriers and facilitators” to “hurdles and facilitators”. Other adaptations included the provision of clear laminated materials as visual aids e.g. pictorial guides for the BORG scale, pain visual analogue scale and treatment flowcharts.
	Intervention Fidelity
How well*: Training*	Evaluation forms are completed by physiotherapists after PROSPER intervention training. Asked to return completed forms anonymously to trial office. All aspects of training delivery and trial materials are evaluated.
How well*: Physiotherapist (Planned)*	Training emphasises adherence to the PROSPER standardised intervention manual. A senior research physiotherapist (HR), responsible for training, conducts quality assurance checks (QA) by observing at least one treatment session with each participating physiotherapist. Performance and adherence to the standardised protocol is judged against pre-defined criteria. Where treatment fidelity is not acceptable, feedback is given and another QA visit is arranged. QA criteria includes ensuring that the physiotherapist demonstrates each exercise with participants.
How well: *Participants*	Intervention adherence: participants are asked to complete and return exercise diaries to record type and amount of exercises performed over the duration of the study. The physiotherapist reviews this exercise diary with the patient at session to monitor adherence and review progress. These diaries are returned to the trial office for analysis once a participant has been discharged.
How well*: (Actual)*	Data on intervention fidelity will be reported with main trial findings.
Additional criteria as per CERT Criteria	
HOW: delivery	**Item 7:** Decision rules for progressing the exercise program: Progression of shoulder ROM exercises is decided jointly between the patient and the physiotherapist when they can complete the desired number of repetitions comfortably. For strength, each exercise is assessed by performing 2 repetitions and asking the patient to rate their perceived exertion on the modified BORG scale. If their rated exertion is less than 5, the resistance is progressed.
	**Item 6:** Details of motivation strategies: Motivational interviewing techniques are used provide feedback on the exercise diary, explore implementation intentions, collaboratively set goals, and brainstorm hurdles to exercise.
	**Item 8:** Each exercise is described so it can be replicated e.g. illustrations, photographs: All exercises are described in detail using multiple photographs and descriptive text underneath the photographs.
	**Item 10:** Non exercise components: In addition to the exercise programme, physiotherapists may use manual therapy (massage and cord release) to treat soft-tissue tightness or cording. These techniques can be taught to the patient and/or a relative so that they can be performed at home.
	Item 11: How adverse events that occur during exercise are documented and managed: Any adverse event that occurs as a direct result of the PROSPER exercise intervention will be recorded and reported, and reviewed by the Trial Steering and Data Monitoring Committee (TSC and DMC).

#### Structure of programme

**Fig. 2:**
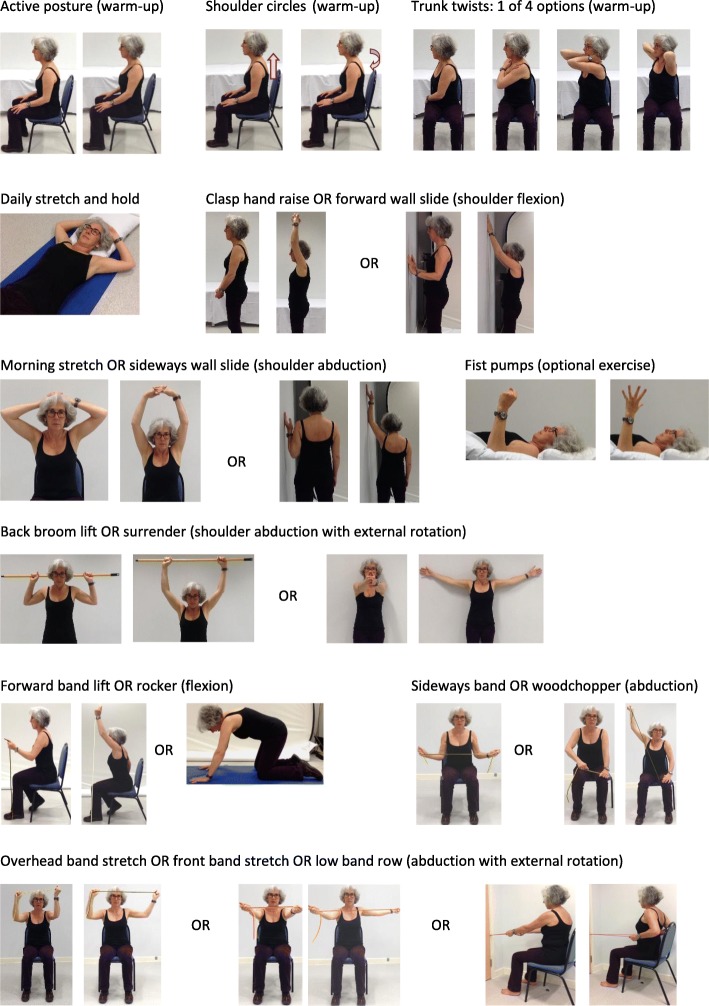
Images of all exercises in the PROSPER intervention

Each trial participant randomised to the PROSPER intervention will be offered **three** face-to-face sessions at set time periods (after surgery at 7–10 days, 4–6 weeks and 12–16 weeks). A further **three** optional appointments can be delivered at any time point and either face-to-face or via the telephone. The first appointment is 60 min to allow time for the initial assessment, with subsequent follow-up sessions lasting 30 min. Each contact will be recorded in a detailed Treatment Log. Tables 1, 2 and 3, and Fig. 2 provide an overview of programme content.

**Table 2:** PROSPER exercise prescription

Exercise type/category		Exercise	Frequency	Sets	Repetitions	Hold	Initial load	Progression
From 7 days after surgery								
Warm-up		Posture check	Twice Daily	1	5	5 s	–	–
		Shoulder circles				n/a		
		Trunk Twists (1–4)				3 s		
Range of Movement	Daily Stretch	Daily stretch & hold	Daily	1 × 10 mins OR 2 × 5 mins				
	Forward	Clasp hand raise OR	Twice Daily	1	5	3 s	–	Step 1: increase up to 10 repetitions Step 2: if applicable, progress to next level of difficulty for the exercise
		Forward wall slide						
	Side	Morning stretch OR						
		Sideways wall slide						
	Open Chest	Back broom lift OR						
		Surrender						
From 4 weeks after surgery								
Strength	Forward	Forward Band Lift	2–3 times every week	1	10 (minimum 8 repetitions; maximum 12 repetitions)	3 s	Selected so that 2 repetitions are rated between 5 and 6 on modified BORG scale	Step 1: maintain 5–6 rating on BORG scale through increasing load (from tan to red to blue theraband tubing). Step 2: Build up to 3 sets with 1–3 min between sets.
		Rocker *(advanced only)*						
	Side	Sideways Band Stretch OR						
		Wood Chopper						
	Open Chest	Over Head Band Stretch OR						
		Front Band Stretch OR						
		Low Band Row						
Physical Activity	*From day 1*	Gentle	Daily	3	10 min	–	–	Build up to 30 mins continuous
	*From 4 weeks*	Moderate	5 times every week	–	30 min			No restrictions after 12 weeks.
	*From 12 weeks*	Moderate to Hard

**Table 3:** Behavioural strategies underpinning the PROSPER intervention

Behavioural strategy	Description
Collaborative goal setting	The physiotherapist helps the participant to set a long-term upper limb functional or PA goal, such as returning to gardening or safely lifting grandchildren. Completing the prescribed exercises are set as a short-term goal; these are then linked to achieving longer-term goals. Ensuring that the participant understands the link between the short and long-term goal is a key part of the adherence strategy.
Confidence scale	Participants are asked to rate their confidence to complete the prescribed exercises on a 10-point Likert scale. If a participant has low confidence (defined as < 7 out of 10 in the Health Trainers Manual), then the physiotherapist will explore reasons for this and will problem-solve solutions to improve confidence in ability to exercise.
Implementation intentions	Participants identify when and where they will complete both their exercises and their exercise diary.
Exercise diary	Participants will complete an exercise diary for review at each appointment. This diary provides immediate feedback and self-monitoring, and serves as a reminder to complete their exercises.
Hurdles and facilitators	At review appointments, any barriers to successful completion of the home exercises are explored. The physiotherapist will help the participant find solutions by identifying factors that can facilitate regular exercise.

#### Participant materials

Each trial participant receives a personal A5 sized folder (‘Your Physiotherapy Folder’) containing coloured pictures and instructions for each exercise, general postoperative advice and details of when and how to seek further help e.g. red flags. Other materials include an exercise dairy and brainstorming crib sheets for identifying hurdles and facilitators to exercise.

#### Session overview

During the first appointment, the physiotherapist records participant details, including current health, previous medical history and exercise/physical activity behaviour. Other assessments include pain intensity, lymphoedema screening (upper limb looking or feeling swollen and/or heavy), visual checks of the surgical wound, posture and active ROM. Short and longer term goals are discussed, along with exploration of intention and confidence in ability to exercise. Where confidence levels are low, hurdles and facilitators are discussed and explored.

At 4 to 6 weeks postoperatively, participants’ shoulder strength is assessed and strength exercises are prescribed. Follow-up sessions focus on postoperative complications, problem solving, self-monitoring, exercise progression and physical activity. Physiotherapists are encouraged to discharge participants within the first 6 months when the participant has met their functional or PA goal/s. However, participants can contact their physiotherapist for advice and support up to 12 months after randomisation.

#### Range of movement and stretching exercises

Participants should always perform a warm-up consisting of active posture correction along with deep breathing, shoulder circles, and trunk twists prior to conducting any shoulder exercises. The intervention targets three movement directions using a combination of active-assisted ROM, active ROM, and stretches: shoulder flexion (forward), shoulder abduction (side), and abduction with external rotation (open chest). There is a menu of **six** ROM exercises to choose from, two in each of the targeted movement directions, with varying degrees of difficulty. Only one ROM exercise from each movement direction is selected and each exercise is repeated five times, twice a day. All participants are asked to do a ‘daily stretch and hold’ of the pectoralis muscle that is held for 10 min (or twice for 5 min) every day. If a participant has suspected lymphoedema, physiotherapists can prescribe an additional exercise (opening and closing fingers into a fist five times every 2 h, ensuring the hand is above the elbow) as recommended by Breast Cancer Care [42].

##### Establishing baseline level and progression for ROM exercises

During the first appointment, three of the six ROM exercises are selected, taking into account patient preference and the physiotherapist’s assessment. All decisions are jointly agreed. Table 2 details the initial target repetitions and exercise frequency; all prescription details, including adaptations, are recorded in the Treatment Log. Progression is achieved by moving to the next level for the chosen exercise, and by increasing repetitions and sets.

#### Strength exercises (from four weeks onwards)

The PROSPER intervention has **seven** strength exercises, each targeting one of the specified movement directions: shoulder flexion, abduction, and abduction with external rotation. Isometric shoulder strength is assessed from 4 weeks after surgery using a standardised protocol. Based upon clinical assessment and participant preference, the physiotherapist and participant jointly select three strength exercises from the menu. Three different resistance bands (Thera-band© tubing) are offered. Each band is cut to one-metre length and provides resistance of: tan (1.1 kg at 100% elongation), red (1.7 kg at 100% elongation) and blue (2.6 kg at 100% elongation).

##### Establishing baseline level and progression for strength exercises

A modified Borg scale is used to determine the correct level of resistance based on self-perceived effort. This 10-point scale has been validated for use in determining intensity of resistance exercises [43]. For each selected strength exercise, participants perform two repetitions on their operated side, then are asked to rate their perceived level of exertion. Target resistance is reached when participants rate their level of exertion as a five or six on the modified scale [43]. While we provide initial prescription and progression recommendations for the strength exercises (Table 2), physiotherapists can tailor this based on individual ability.

#### Physical activity

The physiotherapist will collaborate with the participant to develop a progressive plan to increase physical activity, accounting for individual stage of treatment trajectory and any potential hurdles to achieving the exercise behaviour. The aim is to achieve 150 min of moderate physical activity per week. This can be divided into 10 min, three times a day, five times a week [25].

#### Behavioural support strategies

The following behavioural support strategies are integral components of the final PROSPER intervention [38]. These are implemented using a motivational interviewing approach (Table 3):

(i) **Collaborative goal setting:** The physiotherapist helps the participant to set a long-term upper limb functional or PA goal, such as returning to gardening or safely lifting grandchildren. Completing the prescribed exercises are set as a short-term goal; these are then linked to achieving longer-term goals. Ensuring that the participant understands the link between the short and long-term goal is a key part of the adherence strategy.
(ii) **Confidence scale:** Participants are asked to rate their confidence to complete the prescribed exercises on a 10-point Likert scale. If a participant has low confidence (defined as < 7 out of 10 in the Health Trainers Manual), then the physiotherapist will explore reasons for this and will problem-solve solutions to improve confidence in ability to exercise.
(iii) **Implementation intentions:** Participants identify when and where they will complete both their exercises and their exercise diary.
(iv) **Exercise diary:** Participants will complete an exercise diary for review at each appointment. This diary provides immediate feedback and self-monitoring, and serves as a reminder to complete their exercises.
(v) **Hurdles and facilitators:** At review appointments, any barriers to successful completion of the home exercises are explored. The physiotherapist will help the participant find solutions by identifying factors that can facilitate regular exercise.

#### Manual therapy

A standardised protocol for two manual therapy techniques is included in the PROSPER intervention: (i) gentle massage using effleurage and petrissage and (ii) cord release using a traction method. These techniques are optional for patients with suspected cording that is painful and/or restricting ROM. Wherever possible, the focus is on instructing the patient and their partner on how to self-manage using these techniques at home.

#### Management of complications

The PROSPER intervention provides guidance and treatment pathways for when to refer participants to other services e.g. breast care nurses, surgical team, general practitioner (GP) and/or lymphoedema specialist. Concerns requiring onward referral include suspected wound infection, persistent seroma, severe postoperative pain, and lymphoedema. We also provide general advice on cancer-related fatigue and activity pacing.

## Discussion

In accordance with the MRC framework, we have described the multi-phased development of a complex exercise intervention with integrated behavioural strategies. All phases were important for the production of a fully manualised, structured intervention suitable for evaluation in a large-scale multicentre pragmatic RCT (ISRCTN: 35358984). The full PROSPER intervention manual and related materials will be available for wider access on completion of the main trial, according to funder and institutional repository requirements. The trial recruited the first participant in January 2016 and follow-up is ongoing until July 2018. Qualitative interviews will be conducted with participating physiotherapists on completion of intervention delivery; to date, physiotherapist engagement has been very positive. Study findings will be reported in 2019.

## Abbreviations

ANC: Axillary Node Clearance
AROM: Active Range of Movement
BC: Breast Cancer
BCC: Breast Cancer Care
CERT: Consensus on Exercise Reporting Template
CI: Confidence Interval
DASH: Disability of the Arm Shoulder and Hand scale
HTA: Health Technology Assessment
MI: Motivational Interviewing
NIHR: National Institute for Health Research
NHS: National Health Service
PA: Physical Activity
PPI: Patient and Public Involvement
PROSPER: PRevention Of Shoulder ProblEms tRial
RCT: Randomised Controlled Trial
ROM: Range of Movement
RR: Relative Risk
TIDier: Template for Intervention Development and Replication

## References

[1] CRUK. Cancer Res *UK:* Breast Cancer *Survival* Statistics [cited 2014. Available from: http://www.cancerresearchuk.org/.

[2] McNeely ML, Campbell K, Ospina M, et al. Exercise interventions for upper-limb dysfunction due to breast cancer treatment. The Cochrane database of systematic reviews 2010(6):CD005211. 10.1002/14651858.CD005211.pub2 PMC12861582 20556760

[3] Mejdahl MK, Andersen KG, Gärtner R (2013). Persistent pain and sensory disturbances after treatment for breast cancer: six year nationwide follow-up study. BMJ.

[4] (MRC) MRC. Developing and Evaluating Complex Interventions New Guidance. www.mrc.ac.uk/complexinterventionsguidance, 2000. Accessed 1 Mar 2015.

[5] Slade SC, Dionne CE, Underwood M, et al. Consensus on exercise reporting template (CERT): a modified Delphi study. Phys Ther. 2016;96(10):1514-24. Epub 2016 May 5. 10.2522/ptj.20150668 27149962

[6] Hoffmann TC, Glasziou PP, Boutron I (2014). Better reporting of interventions: template for intervention description and replication (TIDieR) checklist and guide. Bmj.

[7] Bruce J, Williamson E, Lait C (2018). Randomised controlled trial of exercise to prevent shoulder problems in women undergoing breast cancer treatment: study protocol for the prevention of shoulder problems trial (UK PROSPER). BMJ Open.

[8] Bruce J, Thornton AJ, Powell R (2014). Psychological, surgical, and sociodemographic predictors of pain outcomes after breast cancer surgery: a population-based cohort study. Pain.

[9] Lacomba MT, Sánchez MJY, Goñi ÁZ (2010). Effectiveness of early physiotherapy to prevent lymphoedema after surgery for breast cancer: randomised, single blinded, clinical trial. Bmj.

[10] Lee SA, Kang J-Y, Kim YD (2010). Effects of a scapula-oriented shoulder exercise programme on upper limb dysfunction in breast cancer survivors: a randomized controlled pilot trial. Clin Rehabil.

[11] Kilbreath SL, Refshauge KM, Beith JM (2012). Upper limb progressive resistance training and stretching exercises following surgery for early breast cancer: a randomized controlled trial. Breast Cancer Res Treat.

[12] do Amaral MTP, de Oliveira MMF, Ferreira NO, et al. Manual therapy associated with upper limb exercises vs. exercises alone for shoulder rehabilitation in postoperative breast cancer. Physiother Theory Pract. 2012;28(4):299–306. 10.3109/09593985.2011.604709 22007656

[13] Todd J, Scally A, Dodwell D (2008). A randomised controlled trial of two programmes of shoulder exercise following axillary node dissection for invasive breast cancer. Physiotherapy.

[14] Anderson RT, Kimmick GG, McCoy TP (2012). A randomized trial of exercise on well-being and function following breast cancer surgery: the RESTORE trial. J Cancer Surviv.

[15] Xie X, Liu Z, Qu S (2010). 169 patients with postoperative breast cancer on exercising the function of limbs and investigating quality of life: a clinical study. Chin-Ger J Clin Oncol.

[16] Devoogdt N, Christiaens M-R, Geraerts I (2011). Effect of manual lymph drainage in addition to guidelines and exercise therapy on arm lymphoedema related to breast cancer: randomised controlled trial. Bmj.

[17] Yang EJ, Park WB, Seo KS (2010). Longitudinal change of treatment-related upper limb dysfunction and its impact on late dysfunction in breast cancer survivors: a prospective cohort study. J Surg Oncol.

[18] Levick J. Synovial fluid and trans-synovial flow in stationary and moving normal joints. Joint Loading Biology and health of articular structures Ed Helminen HJ, Kiviranta I, Säämänen AM, Tammi M, Paukkonen K Jurvelin Butterworth & Co Ltd Bristol 1987:150–152.

[19] Box RC, Reul-Hirche HM, Bullock-Saxton JE (2002). Shoulder movement after breast cancer surgery: results of a randomised controlled study of postoperative physiotherapy. Breast Cancer Res Treat.

[20] Stubblefield MD, Keole N. Upper body pain and functional disorders in patients with breast cancer. PMR. 2014;6(2):170–83. 10.1016/j.pmrj.2013.08.605 24360839

[21] Bouffard NA, Cutroneo KR, Badger GJ (2008). Tissue stretch decreases soluble TGF-beta1 and type-1 procollagen in mouse subcutaneous connective tissue: evidence from ex vivo and in vivo models. J Cell Physiol.

[22] Williams PE, Catanese T, Lucey EG (1988). The importance of stretch and contractile activity in the prevention of connective tissue accumulation in muscle. J Anat.

[23] Leong DP, Teo KK, Rangarajan S (2015). Prognostic value of grip strength: findings from the prospective urban rural epidemiology (PURE) study. Lancet.

[24] Christensen JF, Jones L, Andersen J (2014). Muscle dysfunction in cancer patients. Ann Oncol.

[25] Schmitz KH, Courneya KS, Matthews C (2010). American College of Sports Medicine roundtable on exercise guidelines for cancer survivors. Med Sci Sports Exerc.

[26] Klassen O, Schmidt ME, Ulrich CM, et al. Muscle strength in breast cancer patients receiving different treatment regimes. J Cachexia Sarcopenia Muscle. 2017;8(2):305-16. 10.1002/jcsm.12165 PMC5377413 27896952

[27] Strasser B, Steindorf K, Wiskemann J (2013). Impact of resistance training in cancer survivors: a meta-analysis. Med Sci Sports Exerc.

[28] Campbell KL, Neil SE, Winters-Stone KM (2012). Review of exercise studies in breast cancer survivors: attention to principles of exercise training. Br J Sports Med.

[29] Eyigor S, Kanyilmaz S (2014). Exercise in patients coping with breast cancer: an overview. World J Clin Oncol.

[30] Cramp F, Byron-Daniel J (2012). Exercise for the management of cancer-related fatigue in adults. Cochrane Database Syst Rev.

[31] Ballard-Barbash R, Friedenreich CM, Courneya KS (2012). Physical activity, biomarkers, and disease outcomes in cancer survivors: a systematic review. J Natl Cancer Inst.

[32] Mason C, Alfano CM, Smith AW, et al. Long-term physical activity trends in breast cancer survivors. Cancer Epidemiol Prev Biomarkers 2013:cebp 0141.2013. 10.1158/1055-9965.EPI-13-0141 PMC3688258 23576689

[33] Courneya KS, Segal RJ, Gelmon K (2007). Six-month follow-up of patient-rated outcomes in a randomized controlled trial of exercise training during breast cancer chemotherapy. Cancer Epidemiol Prev Biomarkers.

[34] Henriksson A, Arving C, Johansson B (2016). Perceived barriers to and facilitators of being physically active during adjuvant cancer treatment. Patient Educ Couns.

[35] Organisation WH (2004). Global strategy on diet, Phys Act Health.

[36] Romero SA, Li QS, Mao JJ (2017). Factors and barriers associated with changes in physical activity after cancer diagnosis. J Clin Oncol.

[37] Husebø AML, Karlsen B, Allan H (2015). Factors perceived to influence exercise adherence in women with breast cancer participating in an exercise programme during adjuvant chemotherapy: a focus group study. J Clin Nurs.

[38] National Institute for Health and Care Excellence. Behaviour change: individual approaches.(Public health guideline PH49). 2007. Available at: https://www.nice.org.uk/guidance/ph49. Accessed 15 Apr 2017.

[39] Husebø AM, Dyrstad SM, Søreide JA (2013). Predicting exercise adherence in cancer patients and survivors: a systematic review and meta-analysis of motivational and behavioural factors. J Clin Nurs.

[40] Spencer JC, Wheeler SB (2016). A systematic review of motivational interviewing interventions in cancer patients and survivors. Patient Educ Couns.

[41] Fourie W, Robb K (2009). Physiotherapy management of axillary web syndrome following breast cancer treatment: discussing the use of soft tissue techniques. Physiotherapy.

[42] BCC. Breast Cancer Campaign [Available from: http://www.breastcancercare.org.uk/. Accessed 1 Mar 2015.

[43] Buckley J, Borg G. Borg Scales in Strength Training; from theory to practice in younger and older adults. Appl Physiol Nutr Metab. 2011;36(5):682–92. 10.1139/h11-078 21977913

